# The Food Bank of Madrid: A Linear Model for Optimal Nutrition

**DOI:** 10.3390/ijerph17218097

**Published:** 2020-11-03

**Authors:** Rosendo Castañón, Fco. Alberto Campos, Salvador Doménech Martínez, José Villar

**Affiliations:** 1 Institute for Research in Technology IIT, ICAI, Universidad Pontificia Comillas, 28015 Madrid, Spain; Alberto.Campos@iit.comillas.edu (F.A.C.); Salvador.Domenech@iit.comillas.edu (S.D.M.); 2 INESC TEC—Institute for Systems and Computer Engineering, Technology and Science, 4200-465 Porto, Portugal; jose.villar@inesctec.pt

**Keywords:** nutrition economics, resource optimization, food bank, nutrition in low-income scenarios, COVID-19 pandemic

## Abstract

This work proposes a mathematical linear programming model that addresses the food provisioning problem of the food bank of Madrid. It aims to determine the most appropriate weekly decisions to meet the macro-nutritional requirements of the beneficiaries of this social service, by minimizing the total cost considering third-party donations. The model has been applied to a realistic case study considering a sociological structure of beneficiaries categorized by age and gender and representing the first decile of incomes of the Spanish population. The demand of macronutrients is satisfied by means of nine different groups of food, used to provide some level of variability in the consumption patterns of the beneficiaries. The results provide insight on cost-cutting opportunities related to centralizing the decision-making process, indicating a 10% reduction both in provisioning costs and food quantities. This suggests that the proposed model might serve as a tool for designing new strategies for the provisioning or evaluation of economic and social support policies for the food bank of Madrid.

## 1. Introduction

The COVID-19 pandemic places Spanish society at a juncture in which both the health system and the social conscience are put to the test while the economy falters. In this context, the coming months will likely be marked, among other considerations, by an enormous effort to foresee and limit the magnitude of the economic recession that is coming (and whose effects are already beginning to be glimpsed) on the different groups that make up the society. In a democratic, human, and socially responsible environment, it is relevant to pay special attention to those groups that, during recessions, may find themselves in a situation of greater urgency or economic need. These groups will be, precisely, those who will find themselves in a situation of greater defenselessness and exclusion, since they do not have sufficient means to fight the recession on their own. This situation might even promote certain social destabilization that affects the rest of the groups, in case that the appropriate means are not deployed to help covering their basic needs.

Among other organizations, food banks are key organizations to guarantee the human right to adequate food, by aiming, among other duties, at the distribution of the food to those who have difficulty to avoid hunger, especially important in crisis episodes such as the current COVID-19 pandemic. Indeed, there has been a further increase in the number of individuals requesting help from food banks (from 130 k to 190 k in a 2-month window [1]). Furthermore, the incoming recession places donations to food banks at a risk of de-stabilization (most food donations come from the private sector such as individuals or even solidary companies). In this regard, Madrid’s foodbank has expressed concerns on the expected difficulty in feeding the needy over the coming months. Rapidly rising demand, a possible decrease in donations, and a lack of basic nutrients in the packages the beneficiaries receive are some problems that the food bank is currently facing.

Some models have been proposed in the literature to address decision making under the above-mentioned problems. In the literature, the question of what combination of foods can provide optimal nutrition at the lowest monetary cost, taking into account donations and demand, has usually been addressed with mathematical programming models (see [2,3,4] for linear approaches and [5,6] for a nonlinear case). One of the most interesting conclusions drawn from them is that the models tend to encourage the purchasing foods with high energy density, i.e., with lipids, sweets, and cereals. Actually, this is precisely the usual eating habits of population groups with lower socioeconomic status, just as discussed in [7].

For the case of Spain, there are notable collaborations with the Spanish Federal Organization of Food Banks (FESBAL), such as the one established by the Polytechnic University of Madrid (UPM), through its Chair in Aid to Food Banks [8]. Among the works carried out in this chair, the work entitled “Nutritional Needs of the Spanish Population Belonging to the First Decile of Income” stands out. It is a starting study for the planning of the distribution of food banks. In it, the authors start from the hypothesis that the food banks’ beneficiaries correspond to the first decile of income of the Spanish population. It is a reasonable and sensible hypothesis, which has also been assumed in this paper. The nutritional needs proposed by the UPM study are based on analyzing the energy requirements of different population groups and then imposing ranges of macronutrient intake that fit within the Acceptable Macronutrient Distribution Ranges (AMDRS [9]). However, the UPM study is not taking into account the origin of these macronutrients (it is not considering which food groups should be used to cover the needs), nor it is making a sufficiently precise or detailed representation of the macro-nutritional needs of the different population groups. Moreover, it does not address the economic aspect of the food groups, resulting, consequently, in a study with areas for improvement, which have been addressed in this paper.

This paper addresses the decision making of the Madrid Food Bank to improve its response to the nutritional needs of the different population groups that are beneficiaries of its services. It presents a mathematical-financial linear programming model that helps to make an optimized food supply based on the minimization of the cost that allows to cover the nutritional requirements needed by the beneficiaries. More precisely, the model computes the weekly purchase of several diverse food groups (a total food quantity of each food group, in kg, with its corresponding cost, given by the wholesale market price) that has to be done, after considering third-party donations, in order to satisfy the beneficiary requirements. As relevant cost cutting opportunities emerge when using the model, it might serve as a tool for designing new strategies for the provisioning or evaluation of economic and social support policies for the food bank. It is also a novel contribution in the field of the application of mathematical programming models in the volunteer sector in Madrid (there is no evidence of any similar model available today).

The model has several purposes:

- Allow computing the weekly investment basket to be made to match the nutritional needs of the beneficiaries with the available economic resources.
- Identify supply strategies that reduce the nutritional gap of the disadvantaged population from a precarious situation to values recommended by international organizations. In this sense, the model might serve as a basis for evaluating food policies.
- The potential evaluation of the performance of charitable organizations that intend to satisfy the nutritional needs of the disadvantaged.
- It can also be useful as a complement to other models that face other tasks in the value chain of this type of organization, such as storage or distribution.

The next section describes the methodology followed as well as a graphic representation of the conceptual schema of the work hereby presented, while the proposed optimization model with its main hypotheses are presented in Section 3. Section 4 describes the main results of a realistic case study, and conclusions are drawn in Section 5.

## 2. Methodology

The proposed methodology consists of the following steps:

(1) Review of nutritional requirements by Spanish population groups. Population groups are characterized in this paper by ages, gender, and income ranges.
(2) Search for the nutritional contribution of different food groups to meet the nutritional needs of the different population groups identified in Step 1.
(3) Obtain the price associated with each of the nutritional groups per kg of food group identified in Step 2.
(4) Minimize purchasing costs (mentioned in Step 3) and costs per kg of non-supplied nutrient.

**Figure 1:**
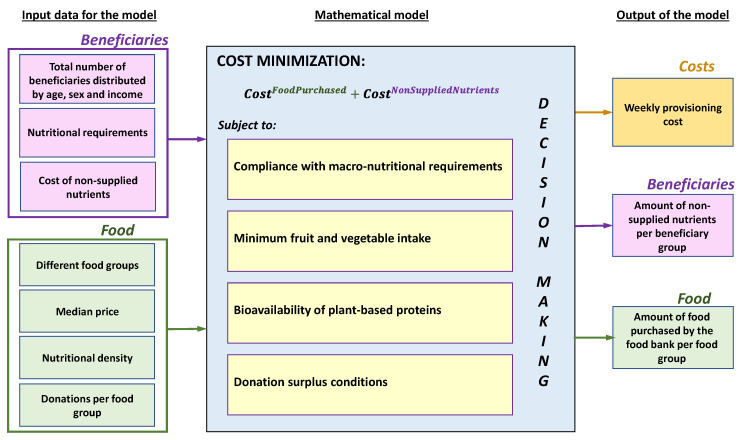
Conceptual schema of the model.

The resulting model can be visually explained through the Figure 1:

## 3. Optimization Model

### 3.1. Modeling Hypotheses

This section describes the hypotheses that have been assumed in the proposed model. Four types of hypotheses are formulated: (1) the nutritional hypotheses made on the nutritional requirements; (2) the economic hypotheses related to the costs of the food groups; (3) the operational hypotheses related to planning and time-dependent decisions; and (4) the execution hypotheses designed for computational reasons or to simplify the analyses to be carried out.

#### 3.1.1. Nutritional Hypotheses

- Instead of using specific products to satisfy the needs of the beneficiaries, general food groups have been used.
- Nutritional requirements per food group are average values from the Recommended Dietary Allowances (RDA [10]). Wherever RDA data are unavailable, they are estimated with the lower ranges proposed in the AMDRS.
- Macronutrient and energy needs are the only nutritional requirements to be considered, leaving aside (i) micro-nutritional and (ii) water requirements. These hypotheses are, respectively, due to (i) the difficulty of finding average or median contributions of micronutrients per food group and (ii) that water is not a scarce resource in the Spanish economy.
- In order to circumvent the lack of micronutrient requirements, the model requires minimum consumptions of fruits and vegetables per day, as recommended by international health organizations (see [11,12]).
- The effect of animal proteins on the bioavailability ([13]) of vegetable proteins is considered, since the successful digestion of vegetable-based proteins requires a minimum intake of micronutrients naturally present in animal-based proteins (such as B12).

#### 3.1.2. Economic Hypotheses

- The cost minimization is carried out from the point of view of the food bank, which is understood as an organization of a social and charitable nature whose purpose is to help satisfy the population’s relevant nutritional requirements.
- The prices used per kg of food are median prices per kg and food group (corresponding to the central position of the price list of the different products considered in each food group). Median costs have been preferred instead of average costs in view of the results of [14], since, according to this study, average costs are much higher than median costs. The reason behind this is that a segment of the population from the middle or upper class can afford “premium” food at very high prices, increasing the average prices, and thus making them less representative of prices the food bank may afford.
- The price of food purchases does not include the cost of additional storage, distribution or manipulation needed after its purchase, nor the cost of the resources (electricity, gas or water) needed for its preparation (such as cooking).
- Costs associated with the purchase of food that is delivered as donations by third-party agents are omitted, since those are sunk costs for the optimization.
- There may be non-supplied amounts of nutrients, which are penalized in the cost function to minimize them. Specifically, this penalty should represent the social cost caused by the non-satisfaction of a nutritional requirement within a given population group. For example, in the case of a lack of essential fatty acids, the penalty should include, among others, the cost associated with the treatments required to treat the diseases due to this deficit, such as obstructions in blood flow, liver diseases, etc. In addition, this penalty can take a higher or lower value depending on whether the group population is of greater or lesser risk, distinguishing, for example, between minors and people in intermediate ages.
- There might be surpluses in donations when their consumption would be detrimental to health. They could even generate, in practice, undesirable costs for storage, in case these costs were represented.

#### 3.1.3. Operational Hypotheses

- As previously explained, donations refer specifically to food delivered to the food bank by solidary third parties that have no information on the final beneficiaries. These food donations are then sorted in the food bank and might be distributed downstream, among the different beneficiary groups, to tackle with, for instance, differences in the quality of the nutrients given by purchased or donated food. Future references to donations in the paper refer to “effective” donations, which are the donations that remain once food in a bad state (because they were in a bad condition at the time they were donated, or because they perished at some point in the food bank’s value chain) is subtracted from the total amount.
- The model considers a dispatch of nutritional needs for a single week. The purchase is then made only once a week, at the beginning, and should cover the needs of the whole week. When modelling a single week, all donations made during a whole year are given in a continuous way, distributed evenly throughout all the weeks of the year. This is equivalent to saying that donations in any given week are the total donations that occur during a year divided by the number of weeks in the year in question. Although this hypothesis fails to capture the impact of seasonality in dietary patterns and requirements, it allows for a powerful operational representation, which could be of more relevance to a centralized planner dedicated to social services that has limited storage and planning capabilities.

#### 3.1.4. Execution Hypotheses

- Considering continuous variables (food quantities are given in kg) makes it easier to compile and execute the model, compared to working with discrete variables (e.g., the number of rice packages, or cans of food), and does not appreciably worsen the resulting costs, being a sufficiently good approximation to the problem.

### 3.2. Objective Function

The model considers a minimization of the costs associated with the weekly provision of nutritional resources to the different population segments that make up the group of beneficiaries of the food bank. The formulation of the objective function is:

(1) $$Min\left(\underset{a}{\sum }PRE_{a}\cdot c_{a}+\underset{pr}{\sum }PEN_{pr}^{\;}\cdot cns_{pr}\right)$$

where $PRE_{a}$ stands for the median price per kg of a given food group, $c_{a}$ stands for the purchased amount of a given food group, $PEN_{pr}^{\;}$ represents the economic penalty associated to an unmatched nutritional requirement for a beneficiary group and $cns_{pr}$ constitutes the quantity of unmatched nutrient for the particular beneficiary group.

### 3.3. Constraints

This section details the constraints to which the objective function is subject.

#### 3.3.1. Compliance with Nutritional Requirements

The following constraint relates the amount of food purchased and donations to the amount of non-supplied nutrients and the nutritional requirements to be satisfied, for the different population groups.

(2) $$\underset{a}{\sum }s_{ap}\cdot NUT_{ar}\;+cns_{pr}\geq NEC_{pr}\cdot CARD_{p}$$

where $s_{ap}$ is the amount of food of group *a* supplied to a population group *p*, $NUT_{ar}$ corresponds to the quantity of a given macronutrient provided by a kg of a certain food group, $NEC_{pr}$ stands for the nutritional requirements of an individual that belongs to a beneficiary group and $CARD_{p}$ holds the value for the number of individuals within each beneficiary group. Therefore, specifically, the above constraint represents that the sum of purchases and food donations made, multiplied by their nutritional content, and added to the amount of nutrient not supplied, must be sufficient to cover the nutritional needs of the population.

#### 3.3.2. Caloric Limitation from Different Food Groups

There are limits to the amount of energy that can come from the different macronutrients, set within the AMDRS. These limits establish lower and higher levels of the caloric intake of each of these macronutrients, although the constraint only considers the upper bound since the lower bound is already implicitly included in (2).

(3) $$CAL_{r}\cdot \underset{p}{\sum }s_{ap}\cdot NUT_{ar}\leq MCAL_{r}^{\;}\cdot \;NEC_{p,‘ener’}\cdot CARD_{p}$$

where $CAL_{r}$ represents the caloric density of a given macronutrient and $MCAL_{r}$ signifies the maximum allowed contribution of said macronutrient to energy requirements.

This constraint can be applied only to some specific macronutrient groups, for example, those defined within the AMDRS: proteins, lipids, and carbohydrates. 

#### 3.3.3. Food Distribution and Limited Surplus:

The following constraint represents this distribution among the different population groups with the possible surpluses produced.

(4) $$\underset{p}{\sum }s_{ap}=c_{a}+DON_{a}-e_{a}$$

$c_{a}$ represents the total purchased a of food group *a*, $DON_{a}$ is the total amount of donations of said food group and $e_{a}$ quantifies surpluses in donations. In case of surpluses, it might even be convenient to move them to other places in Spain where such food could be scarcer.

Note that if a distinction between purchased and donated food distributed to the final beneficiaries was to be considered in the model, Constraint (4) should be replaced with the following Constraints (5)–(7):

(5) $$\underset{p}{\sum }d_{ap}=DON_{a}-e_{a}$$

(6) $$s_{ap}=c_{ap}+d_{ap}$$

(7) $$c_{a}=\underset{p}{\sum }c_{ap}$$

Ultimately, this alternative formulation untaps the potential of the model to distinguish between the purchased ($c_{ap}$) and donated ($d_{ap}$) food distributed across the different beneficiaries, which could additionally serve to portray other relevant dimensions of stock distribution through, for example, time-related constraints representing expiration dates or storage capabilities, or even geographical characteristics of donations when they are transported to the final beneficiaries.

#### 3.3.4. Minimum Consumption of Fruits and Vegetables

Micro-nutritional contribution of the different food groups can be treated with the same methodology as the macro-nutritional contribution. However, since these contributions are difficult to estimate from the literature, a minimum quantity of fruits and vegetables (which are the main source of micronutrients in the diet) has been established instead, applying the following constraint:

(8) $$\underset{a^{fv}}{\sum }s_{ap}\geq \;QFV_{p}$$

$QFV_{p}\;$ is the minimum amount of fruits and vegetables to be consumed.

#### 3.3.5. Bioavailability Condition of Plant-Based Proteins

When planning a diet, it is not only important to attend to the intake of nutrients, but it is also relevant to pay attention to the mechanisms that would lead to their absorption by the body [15]. Although there is no record of an optimal ratio between the intake of animal and vegetable proteins, it is common to find proposals that, at the very least, establish a minimum quantity *AVSP_p_* of vegetable protein per *g* of animal protein to enhance its bioavailability, i.e.,

(9) $$\underset{a^{A}}{\sum }s_{ap}\cdot NUT_{a,‘protein’}\;\geq AVSP_{p}\cdot \underset{a^{V}}{\sum }s_{ap}\cdot NUT_{a,‘protein’}$$

It is important to note that other studies (see [16]) suggest the opposite relationship, supporting that the amount of animal protein should be upper bounded by the amount of vegetable protein. Those same works usually suggest that a high consumption of animal protein is linked to the development of heart and other diseases. However, in other recent studies ([17,18]) it seems that the negative impact on health of excess animal protein only occur in case of previous pathologies. Since it is rare that the population in need has a surplus of animal protein, it has not been considered necessary to assess whether it was appropriate to implement the above constraint in the opposite direction.

## 4. Case Study

The study of the real operation of the Food Bank of Madrid during the year 2018 is presented in this section. The linear programming problem described in the previous sections has been programmed with GAMS v24.8 [19], and solved using IBM’s CPLEX (Armonk, NY, USA) [20], on a personal computer manufactured by HP, equipped with an Intel i7-6700HQ CPU at 2.6 GHz and 16 GB of DDR3 RAM (Palo Alto, CA, USA). The execution time takes up 3 s.

### 4.1. Inputs

This section sets out the details of the sources and the treatment of the input data of this case study. 

#### 4.1.1. Indexes

- Food groups *a* are: cereal, vegetables, fruit, fats and oils, dairy, fish, meat, eggs and pulses.
- Animal proteins *a^A^* are: dairy, fish, meat and eggs.
- Vegetables proteins *a^V^* are: cereal, vegetable, fruit and legumes.
- Population groups p: men and women from 9 to 75 years of age are studied, leaving out infants, young children and the casuistry of pregnancy (pregnant and lactating women), in line with [21]. The proportion of beneficiaries by age group is taken from the first income decile in Spain, in 2018, according to data from [22].
- Nutrients *r* are: carbohydrates, fibre, lipids, omega-3, omega-6, protein and energy.

#### 4.1.2. Nutrition Requirements ($NEC_{pr}$)

The macro-nutritional requirements by population group are obtained from official sources such as the World Health Organization (WHO) [23] and the Food and Agriculture Organization of the United Nations (FAO) [24], or from medical literature, such as the Harrison’s Principles of Internal Medicine [25] and the National Academies Press [26], since the same figures on minimum macro-nutritional and energy requirements are presented in all of them, reflecting consensus. However, since these works do not provide a minimum dose of lipids, this value was set to the lower range of the AMDRS. On the other hand, the caloric requirements of each group are taken from [27], since it presents a more detailed study of the energy requirements of the different groups. Table 1 presents the data derived from the search in the aforementioned reports, with a mathematical treatment corresponding to the escalation of the requirements on a weekly basis.

To enhance the bioavailability of vegetable proteins, a minimum of 1 g of animal protein for every 3 g of vegetable protein has been established, i.e., *AVSP_p_* = 1/3.

#### 4.1.3. Nutritional Density of Each Food Group ($NUT_{ar}$)

The nutritional density of the different food groups has been obtained from [21] that considers the type of products consumed in Spain, reflecting, on the one hand, the nutritional quality of the food in the territory and, on the other, the consumption habits of the country. However, it should be noted that, due (among others) to the low resolution of the data presented by the study (understood as the absence of sufficient significant figures), when summing up the nutritional contributions per kg of food, the amounts do not often meet one kg of food. For this reason, in this paper the data have been corrected, applying a uniform correction coefficient on the macro-nutritional contributions. The uncorrected and corrected results are presented in Table 2 and Table 3, to illustrate the effect of this correction.

#### 4.1.4. Costs of Each Food Group ($PRE_{a}$)

As mentioned, median costs per food group (in EUR/g) have been considered in this paper. They have been obtained from the work performed by Drewnoski and Darmon, combining [14], that presents the median costs in EUR/100 kcal of the different food groups with [28], which proposes a way to transform EUR/100 kcal into EUR/100 g for each of said food groups. Table 4 presents the cost of each food group.

By simplicity, and in the absence of further studies to estimate the sanitary cost when facing illnesses related to a lack of a particular nutrient, an equal cost $PEN_{pr}^{\;}$ of EUR 1000 per kg of nutrient not supplied has been considered for all population groups. A further (and complex) analysis of the estimation of this penalty is out of the scope of this paper and is proposed as a possible line of research.

#### 4.1.5. Number of Beneficiaries ($CARD_{p}$)

The distribution between population groups given by the first decile of income presented by the Spanish National Institute of Statistic (INE) in [22], has been applied to the total number of beneficiaries of the food bank of Madrid during 2018, which can be obtained from [29]. Table 5 shows the total number of beneficiaries per population group that would be attended over the course of a year. Since only a number of these beneficiaries require the aid of the food bank on a regular basis, it will be considered that the food bank must only cover the needs of the 50% of the whole of the beneficiary population.

#### 4.1.6. Amount of Donations ($DON_{a}$)

The total yearly amount of food donated from particulars and from the European Aid to the Most Deprived (FEAD [30]) in the year 2018, which can be found in [29] (annual report of the food bank of Madrid), has been scaled to a week (assuming 52 weeks in 2018). Table 6 presents the effective food donations, which, as said previously, take into account that some amount of the total donations are not received in good state or that they perish in the initial stages of the supply chain (for example, during storage).

### 4.2. Outputs

**Figure 2:**
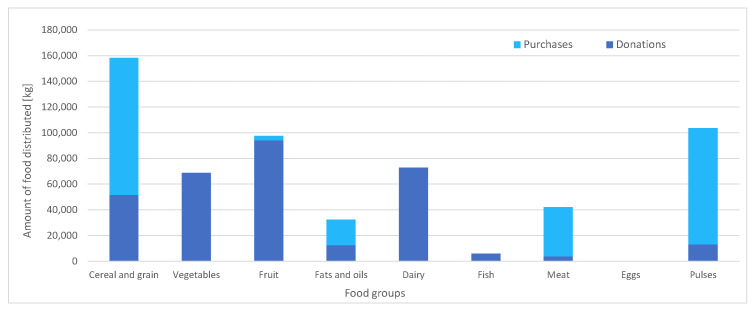
Amounts of distributed food, per food group.

**Figure 3:**
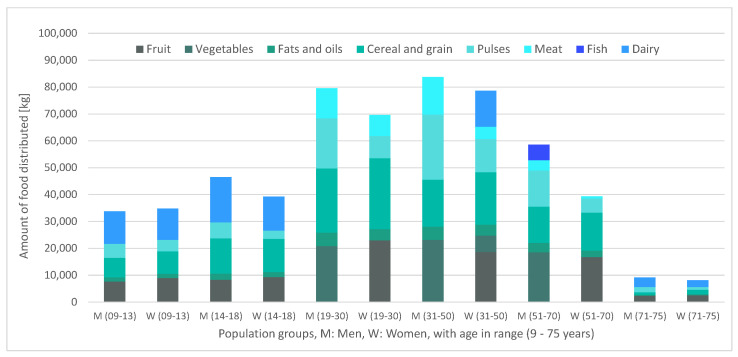
Amounts of distributed food, per food and beneficiary group.

Below are presented, first, in Figure 2, the food distributed for the average week ($\underset{p}{\sum }s_{ap}$), distinguishing purchases ($c_{a}$) from donations (input $DON_{a}$), then, in Figure 3, the specific manner in which this food is distributed among the different population groups, $s_{ap}$.

It can be verified that the aggregate of purchases and donations (Figure 3) responds consistently, since the proportionality between the quantity of food distributed and the number of beneficiaries per population group in Table 5 holds. Regarding the distribution of the food among the beneficiaries, there is a strong presence of the nutritional groups of cereals and pulses throughout all population groups as well as a predominance of meat over fish as a source of animal protein. Figure 2 highlights the role played by dairy and vegetables: the distribution of these food groups occurs only due to donations, and not because of purchases. These donations displace meat and fruit consumption, respectively, which allows for the satisfaction of the minimum consumption of fruits and vegetables and bioavailability condition of plant-based proteins. Another interesting observation is the fact that dairy products are mainly distributed among the minor and the elderly groups and that vegetables are distributed among adults (between 31 and 70 years old), which seems to imply that the nutritional contributions of these foods adjust particularly well to these population groups. Finally, some attention should be drawn to the differences in donations of short-term perishables (fish and meats) vs. non-perishables (such as pulses, or grain). These differences might reflect donators’ perception of a lack of storage capability of the food bank for animal-protein-based food groups, for example, due to an insufficient capacity of preservation technologies, or due to a scarcity of food management professionals. In the case of a strong misalignment between donator perception and the reality of the food bank, it could be beneficial for the latter to devote additional efforts in informing donators of their actual storage capabilities.

**Figure 4:**
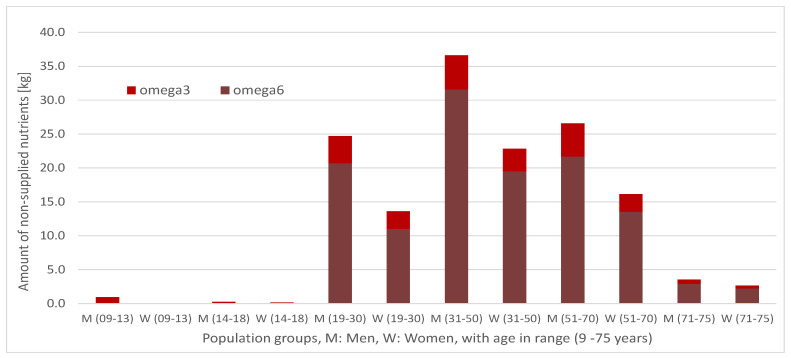
Non-supplied nutrients.

Figure 4 shows the amount of non-supplied nutrients.

In this case, the only non-supplied nutrients are the acid groups omega-3 and omega-6. At this point, it is important to recall that they are precisely those that have the least quantity requirements (see Table 1) and could be therefore intuitively considered to be more essential. However, as previously stated, assigning non-arbitrary penalizations per kg of non-supplied nutrient, would require further research on the impact of their absence in the diet, something that falls beyond the objective of this paper. Either way, the amounts of non-supplied nutrients are truly low, representing only a mismatch of less than 5% with respect to the values of reference, and since nutritional requirements on this paper are based on the RDA metric (a very generous criterion, according to the literature), a slight non-compliance would not be a harmful situation for the health of the vast majority of beneficiaries. The fact that the minor population groups present the lowest values of the amount of non-supplied nutrients seems to indicate that covering the nutritional requirements of these population groups is easier than for the rest (all population groups have the same cost per nutrient not supplied). For this same reason, the results also seem to point out that the distribution of macronutrients required by women is easier to satisfy than that of men. 

Regarding the cost of provisioning, it amounts to kEUR 1364 (kEUR 765 for purchases, and kEUR 599 for donations, which is a sunk cost in the optimization), corresponding to an approximate average cost per person of EUR 70/month (dividing by the number of people and multiplying by the number of weeks in a month). Considering that FESBAL estimates that the cost associated with satisfying a person’s requirements is EUR 60/month, it can be considered that the model is providing very similar results and therefore performs adequately. The difference can be explained by the fact that the model presented here takes into consideration the fulfilment of nutritional requirements in a more exhaustive way, monitoring the satisfaction of nutritional requirements in addition to energy, unlike common charitable organizations, which address famine eradication without consider less urgent aspects such as guaranteeing a minimum amounts of nutrients. 

**Figure 5:**
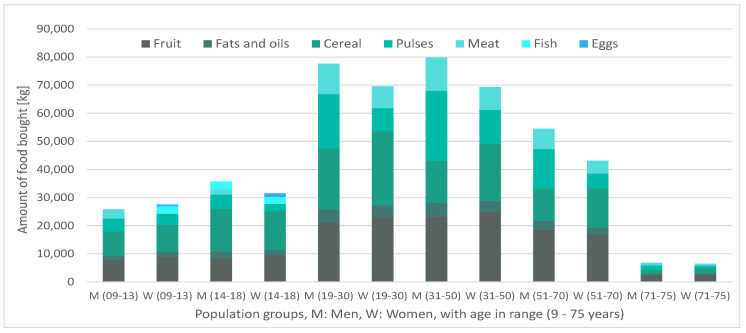
Optimal amounts of food purchased without donations.

Finally, donation surpluses, *e_a_*, takes zero value for all foods and population groups, indicating that donation consumption is not detrimental to health. However, inefficiencies might occur, especially in the sense that the money invested in donations could be better invested in other donated foods or, equivalently, be donated directly as money to FESBAL. The best way to check this is by simulating the case study again, but setting donations to zero, leading to the optimal purchases presented in Figure 5.

Comparing Figure 3 and Figure 5, it can be seen that, by setting donations to zero, the amounts of food to be distributed would be lower (especially in the minor population groups). This suggests that donations might result in unnecessarily large quantities of food, which could increase logistics and storage costs. Moreover, in the case with donations, the total provisioning cost was kEUR 1364, while now, without donations, the total cost is kEUR 1210, which is 11.3% less. In addition, the amounts of food handled in the case with donations versus the case without are 581 tons compared to 528 tons. Therefore, replacing food donations for the corresponding monetary equivalent would favor the acquisition of 9.2% less food quantity (something that might be aligned with lower storage and logistics costs). However, product donations instead of money exchange can appease donators’ fears on the honesty of the management performed by the food back and therefore incentivize donations.

## 5. Conclusions

In this work, a linear mathematical programming model for weekly nutritional supply has been proposed, which, used by the food bank of Madrid, could allow the organization to maximize the social utility of its available resources. The model considers the prices to be paid for different types of foods, and also other cost factors such as health and social ones (like medical treatments costs due to bad nutrition problems).

More specifically, the model minimizes the sum of the costs of purchasing food and non-supplied nutrients. The purchases cover the nutritional requirements of different population groups (configured by sex, age and income range) and are complemented by the resources obtained by the food bank as donations. The main constraints allow one to satisfy the minimum nutritional requirements, provide variety in the diet, and comply with the biological mechanisms of nutrient absorption, among others.

The case studies pointed out possible inefficiencies in the mechanisms of food donation of the Food Bank of Madrid with real 2018 data:

- The distribution of macronutrients required by the minor population is easier to satisfy than that associated with older populations, and that of women is easier to satisfy than that of men.
- The use of a quantitative model to optimize food purchases in an organization such as the food bank of Madrid could help reducing costs by more than a 10%.
- There could be an alignment of objectives between the minimization of provisioning costs and the minimization of logistics costs (understood as warehousing and distribution), since optimizing supplies leads to a reduction in food supplies by 9%. This percentage quantifies the magnitude of inefficiencies of a partially decentralized system (where the food bank obtains relevant food amounts via the donations by third-party agents who do not know the number or beneficiaries) with respect to a centralized system (that has perfect information on beneficiary requirements to the fullest extent).

Future work developments will be oriented to:

- Improve the social and cultural dimension of the approach by the representation of the sociological profiles of the different population groups, including individuals of foreign origin, since they habitually maintain the eating habits of their country and are at a greater risk of exclusion.
- Improve the economic dimension of the beneficiaries that might not have access to electricity and gas, which could lead to the consumption of ready-to-eat foods, which often have the worst nutritional composition, such as ultra-processed foods.
- Develop a more comprehensive chronological framework, which would allow one to consider that a large part of the donations take place only in certain peaks of the year. For example, the so called “Big Pickup” and FEAD operations. This would enable the production of a detailed analysis of other relevant activities in the value chain of the food bank of Madrid, such as storage and external logistics, and would therefore make the resulting model an even more powerful tool to detect strategic opportunities aligned with the 2030 Agenda for Sustainable Development. Adding locational information regarding donations but also storage facilities would complement chronology with valuable data and help to design better operational management strategies, and even alternative marketing strategies in the way donations are promoted.
- Analyze the detrimental impact of the absence (or excess) of the macronutrients studied, as well as of minerals (such as K, Na, Mg …) and vitamins (A, C …) (which in this work have been implicitly considered to be provided by fruit and vegetable consumption) on the beneficiaries, based on biological reasons such as the impact on the glycemic index and sanitary costs. Other nutritional recommendations such as, for example, those given in the NOVA framework [31], could be considered in this analysis.

## Figures and Tables

**Table 1:** Macronutritional and energy requirements.

	[Years]	[kg/Week]						[kcal/Week]
Sex	Age	Carbohydrates	Fibre	Lipids	Linoleic Acid (ω-6)	Alfalinoleic Acid (ω-3)	Protein	Energy
Men	9–13	0.910	0.217	0.389	0.084	0.008	0.238	17,500
	14–18	0.910	0.266	0.529	0.112	0.011	0.364	23,800
	19–30	0.910	0.266	0.436	0.119	0.011	0.392	19,600
	31–50	0.910	0.266	0.389	0.119	0.011	0.392	17,500
	51–70	0.910	0.210	0.319	0.098	0.011	0.392	14,350
	>70	0.910	0.210	0.311	0.098	0.011	0.392	14,000
Women	9–13	0.910	0.182	0.342	0.070	0.007	0.238	15,400
	14–18	0.910	0.182	0.389	0.077	0.008	0.322	17,500
	19–30	0.910	0.175	0.342	0.084	0.008	0.322	15,400
	31–50	0.910	0.175	0.303	0.084	0.008	0.322	13,650
	51–70	0.910	0.147	0.272	0.077	0.008	0.322	12,250
	>70	0.910	0.147	0.264	0.077	0.008	0.322	11,900

The minimum consumption *QFV_p_* of fruits and vegetables per day is set by the World Health Organization at 400 g/day, [5], which is considered here to be somewhat high, due, among other things, to the logistical difficulties related to its storage, transport and preservation (which has not been considered in this study). A value of 286 g/day has been set for *QFV_p_* instead, corresponding to 2 kg/week, which is the current minimum quantity set by the food bank of Madrid.

**Table 2:** Macro-nutritional contribution (without correction).

Food Groups	Carbohydrates [g]	Fibre [g]	Lipids [g]	Linoleic Acid (ω-6) [g]	Alfalinoleic Acid (ω-3) [g]	Protein [g]	Water [mL]	Sum of Contributions [g]	Energy [kcal]
Cereal	577.618	32.239	51.427	6.935	0.433	82.377	232.758	976.419	3155.225
Vegetables	81.235	17.195	2.636	0.216	0.094	16.151	831.503	948.720	416.208
Fruit	107.254	15.374	10.236	1.126	0.104	9.974	684.000	826.838	605.806
Fats and oils	0.725	0.000	983.234	84.566	3.854	0.874	31.796	1016.629	8699.919
Dairy	69.209	0.192	39.697	4.185	0.642	48.233	916.600	1073.931	806.022
Fish	2.161	0.000	54.748	18.279	5.602	131.863	598.335	787.106	1069.889
Meat	3.602	0.000	113.909	27.821	3.224	159.899	659.222	936.632	1777.112
Eggs	0.000	0.000	96.833	7.723	0.729	114.970	734.555	946.357	1313.059
Pulses	415.151	82.418	26.448	4.219	0.036	170.941	258.464	953.422	2814.574

**Table 3:** Macro-nutritional contribution (with correction).

Food Groups	Carbohydrates [g]	Fibre [g]	Lipids [g]	Linoleic Acid (ω-6) [g]	Alfalinoleic Acid (ω-3) [g]	Protein [g]	Water [mL]	Sum of Contributions [g]	Energy [kcal]
Cereal	591.568	33.017	52.669	7.102	0.444	84.367	238.379	1000	3231.424
Vegetables	85.626	18.125	2.778	0.228	0.099	17.024	876.447	1000	438.705
Fruit	129.716	18.594	12.380	1.362	0.125	12.063	827.248	1000	732.678
Fats and oils	0.713	0.000	967.151	83.183	3.791	0.860	31.276	1000	8557.613
Dairy	64.445	0.178	36.964	3.897	0.598	44.913	853.500	1000	750.534
Fish	2.745	0.000	69.556	23.223	7.117	167.528	760.171	1000	1359.269
Meat	3.846	0.000	121.615	29.703	3.442	170.717	703.822	1000	1897.343
Eggs	0.000	0.000	102.321	8.161	0.770	121.487	776.192	1000	1387.487
Pulses	435.433	86.444	27.740	4..425	0..038	179..292	271.091	1000	2952.075

**Table 4:** Median price of different food groups.

Food Group	[EUR/100g]
Cereal	0.270
Vegetables	0.119
Fruit	0.101
Fats and oils	0.317
Dairy	0.240
Fish	0.318
Meat	0.318
Eggs	0.200
Pulses	0.290

**Table 5:** Number of people by population group.

Sociological Groups	[People]
Men aged between 9–13 years	7664
Women aged between 9–13 years	8920
Men aged between 14–18 years	8292
Women aged between 14–18 years	9317
Men aged between 19–30 years	20,905
Women aged between 19–30 years	22,996
Men aged between 31–50 years	23,217
Women aged between 31–50 years	24,784
Men aged between 51–70	18,543
Women aged between 51–70	16,774
Men aged between 70–75	2362
Women aged between 70–75	2563

**Table 6:** Amount of donations.

Food Group	[kg/Week]
Cereal and grain	51,667
Vegetables	68,820
Fruit	93,989
Fats and oils	12,519
Dairy	72,742
Fish	5790
Meat	3712
Eggs	0
Pulses	13,178

## Nomenclature

*A. Sets, subsets and indexes*	
*a*	Food groups
*a^fv^* ⊆ *a*	Fruits and vegetables food groups
*a^A^* ⊆ *a*	Food groups with animal proteins
*a^V^* ⊆ *a*	Food groups with vegetal proteins
*p*	Population groups
*r*	Macronutrients (including energy)
*B. Parameters and scalars (capital letters)*	
*NUT_ar_*	Nutritional contribution [kg/kg or kcal/kg]
*NEC_pr_*	Nutritional requirements [kg/kg or kcal/kg]
*PEN_p,r_*	Cost for non-supplied nutrient [EUR/kg or EUR/kcal]
*PRE_a_*	Price [EUR/kg or EUR/kcal]
*DON_a_*	Donations [kg]
*CARD_p_*	# people [1, ∞)
*CAL_r_*	# of kcal [kcal/kg]
*MCAL_r_*	Maximum calories to be derived from a macronutrient [%]
*QFV_p_*	Minimum quantity of fruits and vegetables to be consumed [kg]
*AVSP_p_*	Minimum amount of animal proteins for the bioavailability of vegetal proteins [kg/kg]
*C. Continuous positive variables (lower letters)*	
*c_a_*	Food purchased by the food bank [kg]
*s_ap_*	Food distributed to beneficiary groups [kg]
*e_a_*	Donation excess [kg]
*cns_pr_*	Non-supplied nutrients [kg or kcal]
